# Nonlinear viscoelastic characterization of bovine trabecular bone

**DOI:** 10.1007/s10237-016-0809-y

**Published:** 2016-07-20

**Authors:** Krishnagoud Manda, Robert J. Wallace, Shuqiao Xie, Francesc Levrero-Florencio, Pankaj Pankaj

**Affiliations:** 1 School of Engineering, The University of Edinburgh, The King’s Buildings, EH9 3DW Edinburgh, UK; 2 Department of Orthopaedics, The University of Edinburgh, Chancellors building, EH16 4SB Edinburgh, UK

**Keywords:** Creep, Recovery, Nonlinear viscoelasticity, Recoverable and irrecoverable strains, Trabecular bone, Schapery model

## Abstract

The time-independent elastic properties of trabecular bone have been extensively investigated, and several stiffness–density relations have been proposed. Although it is recognized that trabecular bone exhibits time-dependent mechanical behaviour, a property of viscoelastic materials, the characterization of this behaviour has received limited attention. The objective of the present study was to investigate the time-dependent behaviour of bovine trabecular bone through a series of compressive creep–recovery experiments and to identify its nonlinear constitutive viscoelastic material parameters. Uniaxial compressive creep and recovery experiments at multiple loads were performed on cylindrical bovine trabecular bone samples ($n = 19$). Creep response was found to be significant and always comprised of recoverable and irrecoverable strains, even at low stress/strain levels. This response was also found to vary nonlinearly with applied stress. A systematic methodology was developed to separate recoverable (nonlinear viscoelastic) and irrecoverable (permanent) strains from the total experimental strain response. We found that Schapery’s nonlinear viscoelastic constitutive model describes the viscoelastic response of the trabecular bone, and parameters associated with this model were estimated from the multiple load creep–recovery (MLCR) experiments. Nonlinear viscoelastic recovery compliance was found to have a decreasing and then increasing trend with increasing stress level, indicating possible stiffening and softening behaviour of trabecular bone due to creep. The obtained parameters from MLCR tests, expressed as second-order polynomial functions of stress, showed a similar trend for all the samples, and also demonstrate stiffening–softening behaviour with increasing stress.

## Introduction

Trabecular bone is an open porous composite cellular solid material from an engineering perspective. The apparent level mechanical properties of this cellular material depend on its heterogeneous microstructure, which varies with age, disease, gender and anatomical site being considered (Keaveny et al. 2001). Bone is known to become more porous with age and due to diseases such as osteoporosis (Rachner et al. 2011). Trabecular bone is anisotropic and principal trabecular orientations vary with anatomical site; it is also recognized that its anisotropic character becomes pronounced with age (Singh et al. 1970). The density of this cellular solid has been related to its time-independent elastic stiffness in a number of studies (Currey 1986; Morgan et al. 2003), and these relations are frequently used in computational models of bone and bone-implant systems (Goffin et al. 2013). It has also been recognized that the response of bone to mechanical loads is, in reality, time dependent (Schoenfeld et al. 1974; Zilch et al. 1980). The study of time-dependent behaviour is of interest in a number of contexts: loosening of orthopaedic implants, non-traumatic fractures due to prolonged load over time, viscoelastic compatibility of synthetic bone substitutes and energy absorption during dynamic loads (Norman et al. 2006; Pollintine et al. 2009; Phillips et al. 2006; Linde et al. 1989).

The time-dependent mechanical behaviour of the trabecular bone has been experimentally investigated via relaxation tests (Schoenfeld et al. 1974; Zilch et al. 1980; Deligianni et al. 1994; Bredbenner and Davy 2006; Quaglini et al. 2009), creep tests (Bowman et al. 1994, 1998; Yamamoto et al. 2006; Manda et al. 2016), and dynamic mechanical tests (Guedes et al. 2006; Kim et al. 2012, 2013). Yamamoto et al. (2006) reported that substantial amount of creep develops in the trabecular bone even at smaller load levels corresponding to physiological activities. It has also been found that the time-dependent response is not linear and varies with the applied stress/strain levels (Bowman et al. 1998; Yamamoto et al. 2006; Quaglini et al. 2009), i.e. it cannot be modelled using linear viscoelasticity. However, none of the above studies quantified the nonlinearity in the time-dependent response of the trabecular bone. Characterizing this nonlinearity in the time-dependent behaviour at apparent level is important from both clinical and engineering perspectives. Such characterization can provide insights into the mechanisms contributing to the creep behaviour of the trabecular bone, improve predictions from finite element modelling of bone and bone-implant systems, and help understand osteoporotic fractures.

Many constitutive equations have been developed for characterizing the nonlinear viscoelastic materials, from single integral (Knauss and Emri 1981; Schapery 1969; Christensen 1980) to multiple integral formulations, see e.g. Findley et al. (1976). The single- integral representations have been the most widely applied theories for different viscoelastic materials and are relatively easy to implement in a numerical scheme. Previous studies have developed methodologies to determine the nonlinear viscoelastic parameters based on single integral formulations for materials with power law time dependence (Lou and Schapery 1971) and with Prony series time dependence (Nordin and Varna 2005; Huang et al. 2011). Both creep data during plateau loading and strain recovery data after unloading in a creep–recovery test at different load levels are required for this analysis. Most of these formulations have been used for materials like asphalt concrete and polymers, and the samples were permitted to fully recover between creep–recovery tests at different load levels. However, it is not known how long trabecular bone takes to recover fully between the tests (Yamamoto et al. 2006; Kim et al. 2012; Pollintine et al. 2009). Therefore, it is necessary to develop a methodology that takes into account any residual strains and permits continuous application of loading and unloading phases at different load levels without the need for resting the sample between the loading cycles.

Therefore, the primary objectives of the study were threefold. First, to experimentally measure the time-dependent behaviour of trabecular bone through uniaxial compressive multiple load creep–recovery (MLCR) experiments. Second, to develop a systemic methodology to estimate the associated material parameters from the MLCR tests. Third, to quantify the nonlinearity associated with varying stress levels using the obtained parameters.

## Materials and methods

### Sample preparation and $\mu \mathrm{CT}$ imaging

Fresh proximal bovine femora, female, under 30 months old when killed, were obtained from a local abattoir and were stored at $-20\,^{\circ }\hbox {C}$ until utilized. The bones were allowed to thaw to room temperature before the femoral heads and trochanters were removed using a hacksaw. Transmission radiographs were then taken to identify the principal direction of trabeculae, and 19 cores (15 from three femoral heads and 4 from two trochanters) were extracted using a diamond core drill bit (Starlite, Rosemont, USA) and marrow was kept intact in all the samples to mimic the realistic situation of bone as closely as possible. The heads and trochanters were kept hydrated while drilling in a custom-made holding clamp to mitigate temperature damage. Once extracted, the cores were examined for the presence of a growth plate, and if found, this was removed during sample preparation. A low-speed rotating saw (Buehler, Germany) was used to create parallel sections. The cylindrical bone samples in total $n = 19$ were of diameter $10.6 \pm 0.1\hbox { mm}$ and mean height of $25.0 \pm 2.7\hbox { mm}$. Brass end-caps were glued to each end of the sample using bone cement (Simplex, Stryker, UK) to minimize end-artefacts during compression testing (Keaveny et al. 1997). Effective length ($22.1 \pm 2.6\hbox { mm}$) of each specimen was calculated as the length of the sample between the end-caps plus half the length of the sample embedded within the end-caps (Keaveny et al. 1997), and this effective length was used in calculating average strains.

Before mechanical testing high-resolution microcomputed tomography ($\mu \hbox {CT}$) scans were taken of each sample using a Skyscan 1172 $\mu \hbox {CT}$ scanner (Bruker microCT, Kontich, Belgium). The following scan parameters were used: voxel resolution 17.22 $\upmu$m, source voltage 100 kV, current $100\,\upmu \hbox {A}$, exposure 1771 ms with a 0.5 mm aluminium filter between the X-ray source and the specimen. Image quality was improved by using two-frame averaging. The images were reconstructed with no further reduction in resolution using Skyscan proprietary software, nRecon V1.6.9.4 (Bruker microCT, Kontich, Belgium). Morphometric analysis was performed using CTAn software (Bruker microCT, Kontich, Belgium), and by considering the whole volume within each sample, the ratio of bone volume to total volume (BV/TV) was evaluated along with other microarchitectural indices: trabecular thickness (Tb.Th), trabecular number (Tb.N), trabecular separation (Tb.Sp) and structure model index (SMI). Homogeneity analysis was performed on each sample by evaluating the above microarchitectural indices in sub-volumes of four $5\times 5 \times 5~\hbox {mm}$ cubes along the length of each sample. Intraspecimen variations of these indices across each sample were found to be less than $\pm 4\,\%$ with respect to the values when whole volume was considered indicating fairly homogeneous nature and uniform bone quality of each sample. A water bath filled with phosphate-buffered saline (PBS) was used around each sample to keep it hydrated at all times during imaging and through all phases of mechanical testing.

### Creep–recovery experiments

**Fig. 1:**
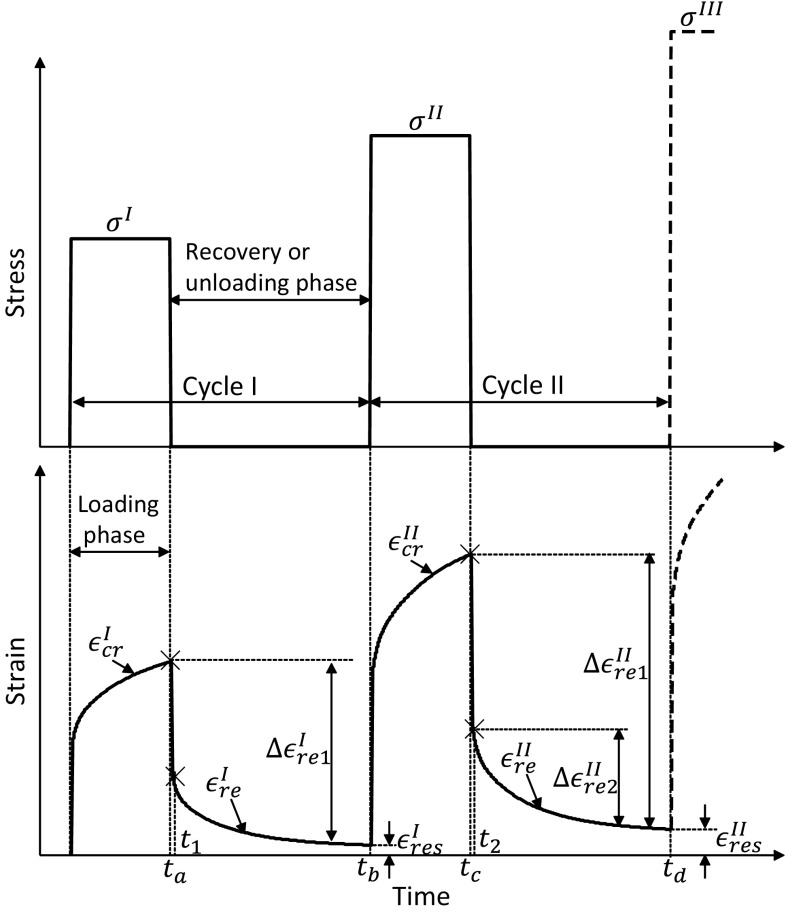
A schematic representation of experimental creep and recovery tests at multiple load levels

Following $\mu \mathrm{CT}$ scanning, each sample was preconditioned by applying 0.1 % apparent strain for ten cycles (Bowman et al. 1994) and was then allowed to recover for 30 min prior to the main mechanical testing. The compressive multiple load creep–recovery (MLCR) experiments as shown in Fig. 1 were conducted on 19 trabecular bone samples using Zwick material testing machine (Zwick Roell, Herefordshire, UK). The trabecular bone macroscopically yields below 0.8 % strains in compression (Kopperdahl and Keaveny 1998; Morgan et al. 2001) in an isotropic manner in strain space (Levrero-Florencio et al. 2016). Therefore, we chose the static strains of 0.2, 0.4, 0.6, 0.8, 1.0, 1.5, 2.0 and 2.5 % in cycles I–VIII, respectively, to measure the time-dependent behaviour at pre- and post-yield regime. These target strains were specified to the Zwick machine in the MLCR tests on each sample which in turn applied the force as a ramp at a strain rate of 0.01 $\text {s}^{-1}$, and when the targeted static strain was reached, a constant load corresponding to this strain was automatically maintained by the machine for 200 s. Each loading step was followed by an unloading step (again at a strain rate of 0.01 $\text {s}^{-1}$) to almost zero (2 N) force, which was maintained for 600 s (see upper part of Fig. 1). This small load of 2 N was to ensure that end-caps remained in contact with the load applicator. The creep deformation was recorded during the loading phase of 200 s and also during the strain recovery (unloading phase) of 600 s for each cycle throughout the experiment for each sample (lower part of Fig. 1). All the tests were load controlled. In our pilot studies, we observed that the creep rate (slope of the creep vs. time curve) becomes constant in less than 200 s during the loading phase (at load levels of interest). Similarly, in the recovery phase, the recovery curves were found to reach a plateau in less than 600 s. Hence, we chose the creep time as 200 s and recovery time as 600 s for all samples in all cycles.

These multiple plateau loads corresponding to above-mentioned static strains were converted to stresses by dividing them with cross-sectional area of each sample. The experiments were stopped if the tertiary creep or failure occurred during the loading phase at any stress level. The tertiary creep or failure was defined as response where creep strain accelerates rapidly and increases beyond 5.0 %. In the following sections, we use the term ‘load’ in Newtons and ‘stress’ in MPa interchangeably, and also a term ‘applied static strain’ which indicates the plateau loads/stresses corresponding to static strains of 0.2, 0.4, 0.6, 0.8, 1.0, 1.5, 2.0 and 2.5 % in the loading cycles I, II, III, IV, V, VI, VII and VIII, respectively.

### Material model

The time-dependent strain response ($\epsilon _\mathrm{tot}\left( t\right)$) of trabecular bone to an applied load is given by

1 $$\begin{aligned} \epsilon _\mathrm{tot}\left( t\right) =\epsilon _\mathrm{nve}\left( t\right) +\epsilon _\mathrm{irrec}\left( t\right) \end{aligned}$$

where $\epsilon _\mathrm{irrec}\left( t\right)$ is the irrecoverable strain response and $\epsilon _\mathrm{nve}\left( t\right)$ is the recoverable nonlinear viscoelastic strain. For linear viscoelastic materials $\epsilon _\mathrm{nve}\left( t\right) =\epsilon _\mathrm{ve}\left( t\right)$ . Uniaxial stress-strain relation, represented by Boltzmann superposition integral (Findley et al. 1976), for linear viscoelastic materials is given by

2 $$\begin{aligned} \epsilon _\mathrm{ve}\left( t\right) =\int _0^t D\left( t-\tau \right) \frac{\mathrm{d}\sigma }{\mathrm{d}\tau }\mathrm{d}\tau \end{aligned}$$

or, equivalently

3 $$\begin{aligned} \epsilon _\mathrm{ve}\left( t\right) =D_0\sigma + \int _0^t {\Delta } D\left( t-\tau \right) \frac{\mathrm{d}\sigma }{\mathrm{d}\tau }\mathrm{d}\tau \end{aligned}$$

where $\sigma$ is an arbitrary stress input, $D(t)=D_0+{\Delta } D(t)$ is the total creep compliance, $D_0$ is instantaneous compliance that describes the elastic response at time $t=0$, and ${\Delta } D(t)$ is the transient creep compliance that evolves with time. In an ideal creep–recovery test, the plateau stress $\sigma$ is applied at time $t=0$ and removed at $t=t_a$ (see the first cycle in Fig. 1). By substituting this step input of stress $\sigma$ into Eq. 3, the resulting creep strain response ($\epsilon _\mathrm{cr}$) during loading phase, $0<t<t_a$, in a typical creep–recovery test is obtained as

4 $$\begin{aligned} \epsilon _\mathrm{cr}\left( t\right) =D_0\sigma + {\Delta } D \left( t\right) \sigma + \epsilon _\mathrm{irrec}\left( t\right) \end{aligned}$$

and the strain response during recovery period ($\epsilon _\mathrm{re}$), $t>t_a$, is given by

5 $$\begin{aligned} \epsilon _\mathrm{re}\left( t\right)= & {} \epsilon _\mathrm{cr}\left( t\right) -\epsilon _\mathrm{cr}\left( t-t_a\right) \nonumber \\= & {} \left[ {\Delta } D\left( t\right) -{\Delta } D\left( t-t_a\right) \right] \sigma +\epsilon _\mathrm{irrec}\left( t_a\right) \end{aligned}$$

It is important to note that it is not possible to perform, in practice, ideal creep–recovery experiments with instantaneous load application at $t=0$. In this study, the load application in MLCR tests was a finite ramp with the strain rate of 0.01 $\mathrm s^{-1}$. We assumed that this strain rate is sufficiently fast to be treated as instantaneous for the range of strains considered in this study; it was, therefore, assumed that it has negligible influence on the results.

Our preliminary experimental analysis revealed that the recoverable behaviour is not linear and is dependent on the applied stress. Also previous studies (Yamamoto et al. 2006; Quaglini et al. 2009) have recognized that the time-dependent behaviour of the trabecular bone is not linear and varies with the applied stress/strain. In order to capture this nonlinearity, the stress-dependent nonlinear viscoelastic models were considered in this study.

Several general constitutive models have been proposed to describe the behaviour of nonlinear viscoelastic materials (Schapery 1969; Christensen 1980; Knauss and Emri 1981). The thermodynamics-based theory using single integral nonlinear viscoelasticity developed by Schapery (1969, (1997), which utilizes the same structure as the linear integral model, has been shown to be a convenient formulation (Smart and Williams 1972). Also, Dillard et al. (1987) compared the Schapery’s model to several other nonlinear viscoelastic formulations and showed that Schapery’s model produces most accurate results for both given stress or strain inputs. It has also been shown that this model is adaptable to many other nonlinear viscoelastic materials, like asphalt concrete (Huang et al. 2011), polymers (Lai and Bakker 1995) and ligaments (Provenzano et al. 2002). It was, therefore, thought to be appropriate for modelling trabecular bone in this study. The nonlinear constitutive parameters in the Schapery’s model conveniently describe the nonlinearities based on stress.

The nonlinear viscoelastic model proposed by Schapery (1969) is given by

6 $$\begin{aligned} \epsilon _\mathrm{nve}\left( t\right) =g_0{D}_0{\sigma }+g_1\int ^t_0 {\Delta } D\left( \psi ^t-\psi ^\tau \right) \frac{\mathrm{d}\left( g_{2} \sigma \right) }{\mathrm{d}\tau } \mathrm{d}\tau \end{aligned}$$

where $g_0$, $g_1$, $g_2$ and $a_\sigma$ are stress-dependent nonlinear viscoelastic (VE) parameters. The parameter $g_0$ is a nonlinear instantaneous compliance parameter that scales the reduction or increase in instantaneous elastic compliance. Transient nonlinear parameter $g_1$ measures the nonlinearity effect in the transient compliance, and the parameter $g_2$ describes the effect of the loading rate on the transient creep response as well, and $\psi ^t$, called reduced time, is given by

7 $$\begin{aligned} \psi ^t=\int ^t_0{\frac{\mathrm{d}\tau '}{a_{\sigma \left( \tau '\right) } a_{T\left( \tau '\right) } a_{e\left( \tau '\right) }}} \end{aligned}$$

where $a_\sigma$, $a_T$ and $a_e$ are stress, temperature and other environment time-shift factors, respectively. In this work, the effects of temperature and other environment variables are not considered, and therefore, $a_T=a_e=1$. For the linear viscoelastic materials, the parameters $g_0=g_1=g_2=a_{\sigma }=1$, such that Eq. 6 reduces to the Boltzmann superposition integral of Eq. 3. The transient compliance in Eq. 6 is represented by Prony series as

8 $$\begin{aligned} {\Delta } D\left( \psi ^t\right) =\sum _{n=1}^{N_{\mathrm{pr}}}{D_n\left[ 1-\exp \left( -\lambda _{n}\psi ^t\right) \right] } \end{aligned}$$

where $N_\mathrm{pr}$ is number of Prony series parameters and $D_n$ is *n*th coefficient of the Prony series associated with the reciprocal of *n*th retardation time, $\lambda _n$. Similar to the Eqs. 4 and 5, the strain responses during loading and recovery phases in a typical creep–recovery test are given by

9 $$\begin{aligned} \epsilon _\mathrm{cr}\left( t\right) =g_0D_0\sigma + g_1g_2{\Delta } D \left( \frac{t}{a_\sigma }\right) \sigma + \epsilon _\mathrm{irrec}\left( t\right) \end{aligned}$$

and

10 $$\begin{aligned} \epsilon _\mathrm{re}\left( t\right) = \left[ g_2\sigma {\Delta } D\left( \frac{t}{a_\sigma }\right) -g_2\sigma {\Delta } D\left( \frac{t-t_a}{a_\sigma }\right) \right] +\epsilon _\mathrm{irrec}\left( t_a\right) \nonumber \\ \end{aligned}$$

and the reduced time in Eq. 7 becomes $\psi ^t=t/a_\sigma$.

### Evaluation of model parameters

After selecting Schapery’s constitutive theory, the numerical values of its associated parameters were obtained in a systematic manner from the MLCR experimental data. Most of the approaches that have been suggested previously (Lai and Bakker 1995; Huang et al. 2011) relied on independent creep–recovery tests in which the samples were allowed to recover fully between the tests at different load levels. In this study, the experiments were performed continuously at multiple stress levels with loading and unloading phases. Consequently, our methodology was required to account for residual strains from the previous loading cycles when evaluating the response of the following loading cycle. A schematic depiction of creep and recovery curves, during loading and unloading phases, respectively, at multiple stress levels is shown in Fig. 1.

The components of total strain during the loading and the recovery phases in the first cycle are given by

11 $$\begin{aligned} \epsilon _\mathrm{cr}^\mathrm{I}\left( t\right)= & {} \left[ g_{0}^\mathrm{I}D_0 \sigma ^\mathrm{I}+g_{1}^\mathrm{I}g_{2}^\mathrm{I}\sigma ^\mathrm{I} {\Delta } D\left( \frac{t}{a_{\sigma }^\mathrm{I}}\right) \right] +\epsilon _\mathrm{irrec}^\mathrm{I}(t) \end{aligned}$$

and

12 $$\begin{aligned} \epsilon _\mathrm{re}^\mathrm{I}\left( t\right)= & {} \left[ g_{2}^\mathrm{I}\sigma ^\mathrm{I} {\Delta } D\left( \frac{t}{a_{\sigma }^\mathrm{I}}\right) \right. \nonumber \\&\left. -\,g_{2}^\mathrm{I}\sigma ^\mathrm{I} {\Delta } D\left( \frac{t-t_a}{a_{\sigma }^\mathrm{I}}\right) \right] +\epsilon _\mathrm{irrec}^\mathrm{I}(t_a) \end{aligned}$$

where superscripts denote the loading cycle number and subscripts to the time variable *t* are different time points in the MLCR test as shown in Fig. 1.

First step in the analysis procedure is to obtain the Prony series coefficients associated with linear viscoelastic response. It was assumed that the trabecular bone behaves in a linear viscoelastic manner until the first loading cycle (or at a lowest stress level corresponding to 0.2 % of static strain) for each sample. Hence, the corresponding nonlinear VE parameters $g_0^\mathrm{I}=g_1^\mathrm{I}=g_2^\mathrm{I}=a_{\sigma }^\mathrm{I}=1$ for the first loading cycle. The irrecoverable strain, in the first cycle, is constant once the load is removed at $t=t_a$, and therefore, by taking the difference between Eq. 11 at $t=t_a$ and Eq. 12, it is possible to eliminate the irrecoverable strain and the remainder gives purely recoverable (viscoelastic) response. Therefore, the viscoelastic recovery strain ${\Delta }\epsilon _\mathrm{re1}^\mathrm{I}$ between $t_a$ and $t_b$ in the first loading cycle is given by

13 $$\begin{aligned} {\Delta }\epsilon _\mathrm{re1}^\mathrm{I}\left( t\right)= & {} \epsilon _\mathrm{cr}^\mathrm{I}(t_{a})-\epsilon _{re}^\mathrm{I}(t)\nonumber \\= & {} g_{0}^\mathrm{I}D_0\sigma ^\mathrm{I} \nonumber \\&+ \left\{ \begin{array}{l} g_{1}^\mathrm{I}g_{2}^\mathrm{I} \sigma ^\mathrm{I} \sum _{n=1}^{N_\mathrm{pr}}D_{n}\left[ 1-\exp (-\lambda _{n}\frac{t_{a}}{a_{\sigma }^\mathrm{I}})\right] \\ -g_{2}^\mathrm{I}\sigma ^\mathrm{I}\sum _{n=1}^{N_\mathrm{pr}}D_{n}\left[ 1-\exp \left( -\lambda _{n}\frac{t}{a_{\sigma }^\mathrm{I}}\right) \right] \\ +g_{2}^\mathrm{I}\sigma ^\mathrm{I}\sum _{n=1}^{N_\mathrm{pr}}D_{n}\left[ 1-\exp \left( -\lambda _{n}\frac{t-t_{a}}{a_{\sigma }^\mathrm{I}}\right) \right] \end{array} \right\} \nonumber \\ \end{aligned}$$

The unknown linear viscoelastic coefficients $D_0$, $D_n$ and $\lambda _n$ ($n=1,2,\ldots ,N_\mathrm{pr}$) were obtained from the first creep–recovery cycle by minimizing the error between the experimental measurements and Eq. 13 using nonlinear least squares fit for each sample. The number of Prony terms, $N_\mathrm{pr}=3$, was found to be sufficient to accurately represent the experimental viscoelastic strain response for all the samples. Also, the viscoelastic recovery compliance in the first cycle was obtained by dividing the ${\Delta }\epsilon _\mathrm{re1}^I$ with $\sigma ^I$.

The total strain components for the second loading cycle, during creep and recovery phases, were obtained as

14 $$\begin{aligned} \epsilon _\mathrm{cr}^\mathrm{II}\left( t\right)= & {} g_{0}^\mathrm{II}D_0\sigma ^\mathrm{II}\nonumber \\&+\,g_{1}^\mathrm{II} \left\{ \begin{array}{l} g_{2}^\mathrm{I}\sigma ^\mathrm{I}{\Delta } D\left( \frac{t}{a_{\sigma }^\mathrm{I}}\right) -g_{2}^\mathrm{I}\sigma ^\mathrm{I}{\Delta } D\left( \frac{t-t_{a}}{a_{\sigma }^\mathrm{I}}\right) \\ +g_{2}^\mathrm{II}\sigma ^\mathrm{II}{\Delta } D\left( \frac{t-t_{b}}{a_{\sigma }^\mathrm{II}}\right) \end{array}\right\} \nonumber \\&+\,\epsilon _\mathrm{irrec}^\mathrm{II}\left( t\right) \end{aligned}$$

15 $$\begin{aligned} \epsilon _\mathrm{re}^\mathrm{II}\left( t\right)= & {} \left\{ \begin{array}{l}g_{2}^\mathrm{I}\sigma ^\mathrm{I}{\Delta } D\left( \frac{t}{a_{\sigma }^\mathrm{I}}\right) -g_{2}^\mathrm{I}\sigma ^\mathrm{I}{\Delta } D\left( \frac{t-t_{a}}{a_{\sigma }^\mathrm{I}}\right) \\ +g_{2}^\mathrm{II}\sigma ^\mathrm{II}{\Delta } D\left( \frac{t-t_{b}}{a_{\sigma }^\mathrm{II}}\right) -g_{2}^\mathrm{II}\sigma ^\mathrm{II}{\Delta } D\left( \frac{t-t_{c}}{a_{\sigma }^\mathrm{II}}\right) \end{array}\right\} \nonumber \\&+\epsilon _\mathrm{irrec}^\mathrm{II}\left( t_{c}\right) \end{aligned}$$

Using the previously known Prony coefficients, the unknown nonlinear VE parameters for second cycle need to be evaluated. In order to achieve this, the irrecoverable strain $\epsilon ^\mathrm{irrec}\left( t\right)$ at $t=t_{c}$ in the second cycle needs to be eliminated by manipulating Eq. 14 and 15. By subtracting the total strain during recovery period $\epsilon _\mathrm{re}^\mathrm{II}\left( t\right)$ from itself at time $t=t_2$, the resulting equation ${\Delta }\epsilon _\mathrm{re2}^\mathrm{II}\left( t\right)$, $t_2<t<t_d$ contains only two unknown parameters $g_2^\mathrm{II}$ and $a_{\sigma }^\mathrm{II}$ as follows:

16 $$\begin{aligned}&{\Delta }\epsilon _\mathrm{re2}^\mathrm{II}\left( t\right) \nonumber \\&\quad = \epsilon _\mathrm{re}^\mathrm{II}\left( t_{2}\right) -\epsilon _\mathrm{re}^\mathrm{II}\left( t\right) \nonumber \\&\quad = g_{2}^\mathrm{I}\sigma ^\mathrm{I}\left\{ \begin{array}{l} \sum _{n=1}^{N_\mathrm{pr}}D_{n}\left[ 1-\exp \left( -\lambda _{n}\frac{t_{2}}{a_{\sigma }^\mathrm{I}}\right) \right] \\ -\sum _{n=1}^{N_\mathrm{pr}}D_{n}\left[ 1-\exp \left( -\lambda _{n}\frac{t_{2}-t_{a}}{a_{\sigma }^\mathrm{I}}\right) \right] \\ -\sum _{n=1}^{N_\mathrm{pr}}D_{n}\left[ 1-\exp \left( -\lambda _{n}\frac{t}{a_{\sigma }^\mathrm{I}}\right) \right] \\ +\sum _{n=1}^{N_\mathrm{pr}}D_{n}\left[ 1-\exp \left( -\lambda _{n}\frac{t-t_{a}}{a_{\sigma }^\mathrm{I}}\right) \right] \end{array}\right\} \nonumber \\&\qquad +\,g_{2}^\mathrm{II}\sigma ^\mathrm{II}\left\{ \begin{array}{l} \sum _{n=1}^{N_\mathrm{pr}}D_{n}\left[ 1-\exp (-\lambda _{n}\frac{t_{2}-t_{b}}{a_{\sigma }^\mathrm{II}})\right] \\ -\sum _{n=1}^{N_\mathrm{pr}}D_{n}\left[ 1-\exp \left( -\lambda _{n}\frac{t_{2}-t_{c}}{a_{\sigma }^\mathrm{II}}\right) \right] \\ -\sum _{n=1}^{N_\mathrm{pr}}D_{n}\left[ 1-\exp \left( -\lambda _{n}\frac{t-t_{b}}{a_{\sigma }^\mathrm{II}}\right) \right] \\ +\sum _{n=1}^{N_\mathrm{pr}}D_{n}\left[ 1-\exp \left( -\lambda _{n}\frac{t-t_{c}}{a_{\sigma }^\mathrm{II}}\right) \right] \end{array}\right\} \end{aligned}$$

These parameters $g_2^\mathrm{II}$ and $a_{\sigma }^\mathrm{II}$ were obtained by minimizing the error between measurements of ${\Delta }\epsilon _\mathrm{re2}^\mathrm{II}$ as shown in Fig. 1 and Eq. 16 using nonlinear least squares method. By taking the difference between the creep strain $\epsilon _\mathrm{cr}^\mathrm{II}\left( t_{c}\right)$ at $t=t_{c}$ and the strain during recovery period $\epsilon _\mathrm{re}^\mathrm{II}\left( t\right)$ at time *t* in the second cycle, the term ${\Delta }\epsilon _\mathrm{re1}^\mathrm{II}$ can be obtained as

17 $$\begin{aligned}&{\Delta }\epsilon _\mathrm{re1}^\mathrm{II}\left( t\right) \nonumber \\&\quad = \epsilon _\mathrm{cr}^\mathrm{II}\left( t_{c}\right) -\epsilon _\mathrm{re}^\mathrm{II}\left( t\right) \nonumber \\&\quad = g_{0}^\mathrm{II}D_0\sigma ^\mathrm{II}\nonumber \\&\qquad +\,g_{1}^\mathrm{II}\left\{ \begin{array}{l} g_{2}^\mathrm{I}\sigma ^\mathrm{I}\sum _{n=1}^{N_\mathrm{pr}}D_{n}\left[ 1-\exp \left( -\lambda _{n}\frac{t_{c}}{a_{\sigma }^\mathrm{I}}\right) \right] \\ -g_{2}^\mathrm{I}\sigma ^\mathrm{I}\sum _{n=1}^{N_\mathrm{pr}}D_{n}\left[ 1-\exp \left( -\lambda _{n}\frac{t_{c}-t_{a}}{a_{\sigma }^\mathrm{I}}\right) \right] \\ +g_{2}^\mathrm{II}\sigma ^\mathrm{II}\sum _{n=1}^{N_\mathrm{pr}}D_{n}\left[ 1-\exp \left( -\lambda _{n}\frac{t_{c}-t_{b}}{a_{\sigma }^\mathrm{II}}\right) \right] \\ \end{array}\right\} \nonumber \\&\qquad -\,\left\{ \begin{array}{l} g_{2}^\mathrm{I}\sigma ^\mathrm{I}\sum _{n=1}^{N_\mathrm{pr}}D_{n}\left[ 1-\exp \left( -\lambda _{n}\frac{t}{a_{\sigma }^\mathrm{I}}\right) \right] \\ -g_{2}^\mathrm{I}\sigma ^\mathrm{I}\sum _{n=1}^{N_\mathrm{pr}}D_{n}\left[ 1-\exp \left( -\lambda _{n}\frac{t-t_{a}}{a_{\sigma }^\mathrm{I}}\right) \right] \\ +g_{2}^\mathrm{II}\sigma ^\mathrm{II}\sum _{n=1}^{N_\mathrm{pr}}D_{n}\left[ 1-\exp \left( -\lambda _{n}\frac{t-t_{b}}{a_{\sigma }^\mathrm{II}}\right) \right] \\ -g_{2}^\mathrm{II}\sigma ^\mathrm{II}\sum _{n=1}^{N_\mathrm{pr}}D_{n}\left[ 1-\exp \left( -\lambda _{n}\frac{t-t_{c}}{a_{\sigma }^\mathrm{II}}\right) \right] \end{array} \right\} \end{aligned}$$

The remaining two parameters $g_0^\mathrm{II}$ and $g_1^\mathrm{II}$ were obtained by minimizing the error between the measurements of ${\Delta }\epsilon _\mathrm{re1}^\mathrm{II}\left( t\right)$ and Eq. 17. By applying the similar procedure to subsequent loading cycles, the associated nonlinear VE parameters were evaluated in all loading cycles. Once all the nonlinear viscoelastic parameters were obtained, the irrecoverable strain response during the loading phase was obtained from Eq. 11 for *N*th cycle as

18 $$\begin{aligned} \epsilon _\mathrm{irrec}^{N}\left( t\right)= & {} \epsilon _\mathrm{cr}^{N}\left( t\right) -\epsilon _\mathrm{nve}^{N}\left( t\right) \end{aligned}$$

where $N=\mathrm{I, II, III,}\ldots = \hbox {loading}$ cycle number. This procedure leads to nonlinear VE parameters that are known at discrete stress levels ($\sigma ^N$), and these parameters can be expressed as functions of stress through interpolation or regression.

### Curve-fitting nonlinear VE parameters

Once all the nonlinear VE parameters were obtained at multiple stress levels, they were fitted with appropriate functions of stress. In this study, we expressed the nonlinear VE parameters as smooth second-order polynomial functions of effective or von Mises stress ($\sigma _\mathrm{eff}$).

19 $$\begin{aligned} g_0= & {} 1+\sum ^{2}_i{\alpha _i{\left\langle \frac{\sigma _\mathrm{eff}}{\sigma _0}-1\right\rangle }^i}\end{aligned}$$

20 $$\begin{aligned} g_1= & {} 1+\sum ^{2}_i{\beta _i{\left\langle \frac{\sigma _\mathrm{eff}}{\sigma _0}-1\right\rangle }^i}\end{aligned}$$

21 $$\begin{aligned} g_2= & {} 1+\sum ^{2}_i{\gamma _i{\left\langle \frac{\sigma _\mathrm{eff}}{\sigma _0}-1\right\rangle }^i}\end{aligned}$$

22 $$\begin{aligned} a_\sigma= & {} 1+\sum ^{2}_i{\delta _i{\left\langle \frac{\sigma _\mathrm{eff}}{\sigma _0}-1\right\rangle }^i} \end{aligned}$$

where

$$\begin{aligned} \left\langle x \right\rangle = {\left\{ \begin{array}{ll} x &{} x>0\\ 0 &{} x\le 0 \end{array}\right. } \end{aligned}$$

**Fig. 2:**
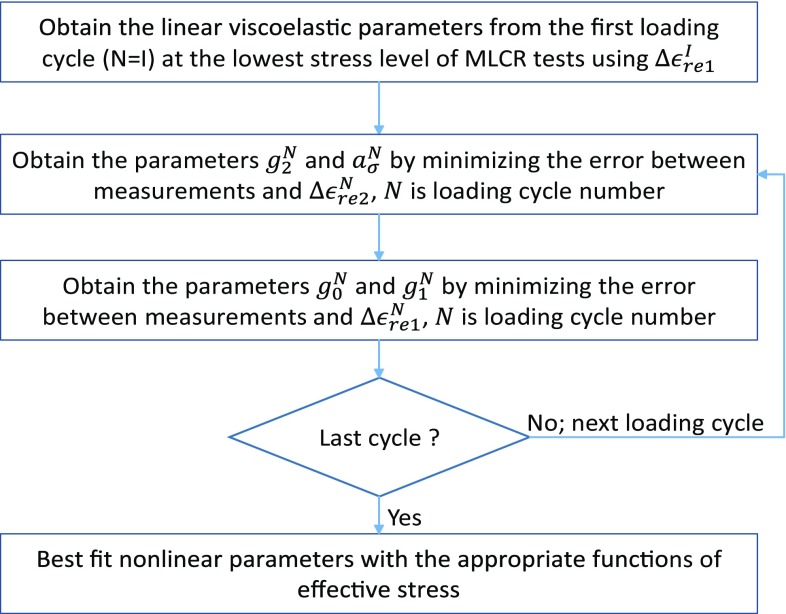
Methodology for estimation of nonlinear viscoelastic parameters of trabecular bone

In our uniaxial MLCR tests, $\sigma _\mathrm{eff}$ is equal to the applied uniaxial stress in each loading cycle. The coefficients $\alpha _i$, $\beta _i$, $\gamma _i$ and $\delta _i$ ($i=1, 2$) were evaluated by fitting the Eqs. 19–22 to the obtained values of the parameters $g_0$, $g_1$, $g_2$ and $a_\sigma$, respectively, in all loading cycles of MLCR tests on each trabecular bone sample. $\sigma _0$ (or $\sigma ^\mathrm{I}$) is the stress in the first loading cycle where linear viscoelastic parameters were determined for each sample. The above methodology for identification of nonlinear viscoelastic parameters is shown concisely as a flowchart in Fig. 2.

**Fig. 3:**
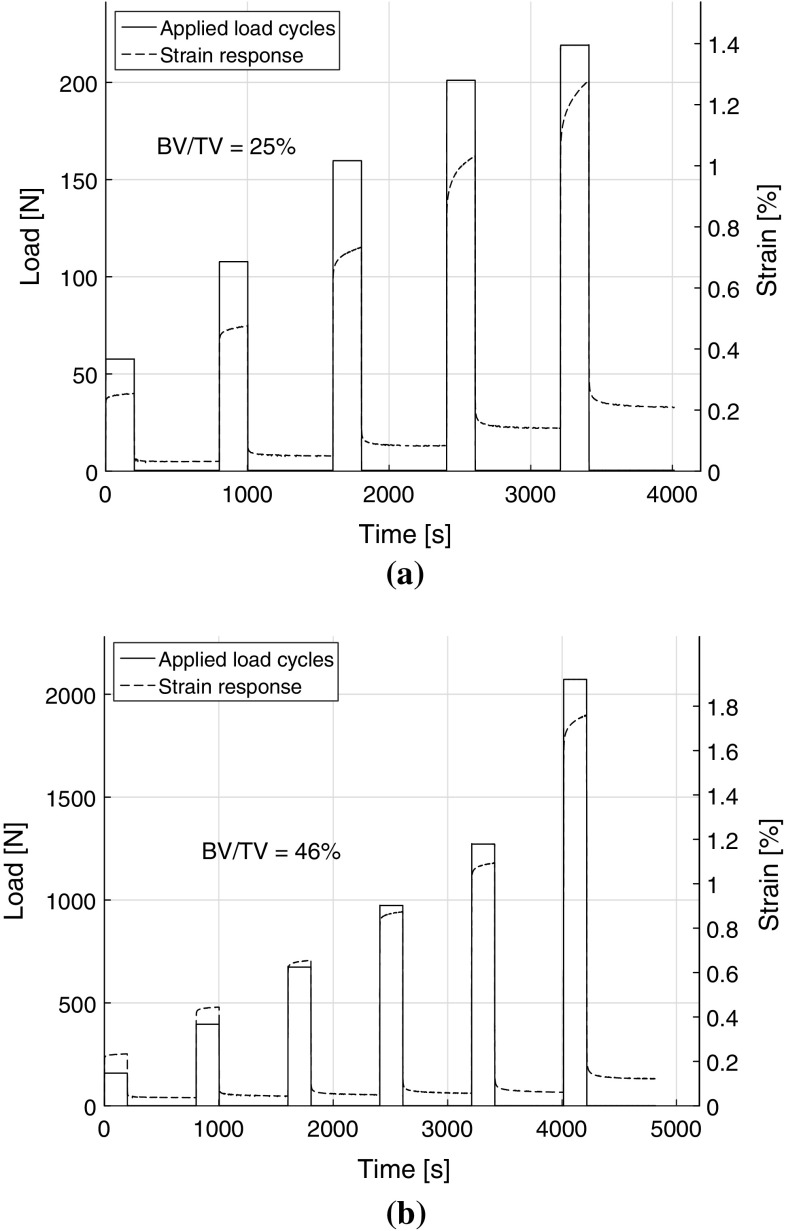
Experimental creep–recovery responses from MLCR tests along with the applied load levels on two typical samples of **a** BV/TV = 0.25 and **b** BV/TV = 0.46. In each cycle, plateau load was held constant for 200 s and strain recovery was measured for another 600 s. The load or stress levels in each of the loading cycles I, II, III, IV, V and VI correspond to the static strains of 0.2, 0.4, 0.6, 0.8, 1.0 and 1.5 %, respectively

## Results

### MLCR experimental data

A total of 19 samples were subjected to MLCR tests and the range of BV/TV of the bone samples was 0.15–0.54. As discussed earlier, our methods involved application of stress corresponding to eight different strain levels. Out of the 19 samples tested, four failed (started displaying tertiary creep) in loading cycle VI, four in loading cycle VII and nine in loading cycle VIII. Only two samples survived all eight stress levels. Typical creep–recovery responses along with the applied load cycles for two samples are shown in Fig. 3. These samples had a BV/TV of 0.25 and 0.46, and were consequently named as S25 and S46. Five cycles of loading (each followed by unloading) with the stress magnitudes of 0.64, 1.19, 1.77, 2.23 and 2.43 MPa were applied to S25, and similarly, six cycles with stress magnitudes of 1.75, 4.38, 7.45, 10.76, 14.06 and 22.92 MPa were applied to S46 as shown in Fig. 3a, b, respectively. The last cycle in each sample where tertiary creep or failure was observed was omitted in the analysis and also not shown in the figures. Results for all samples are provided in Table 1.

**Fig. 4:**
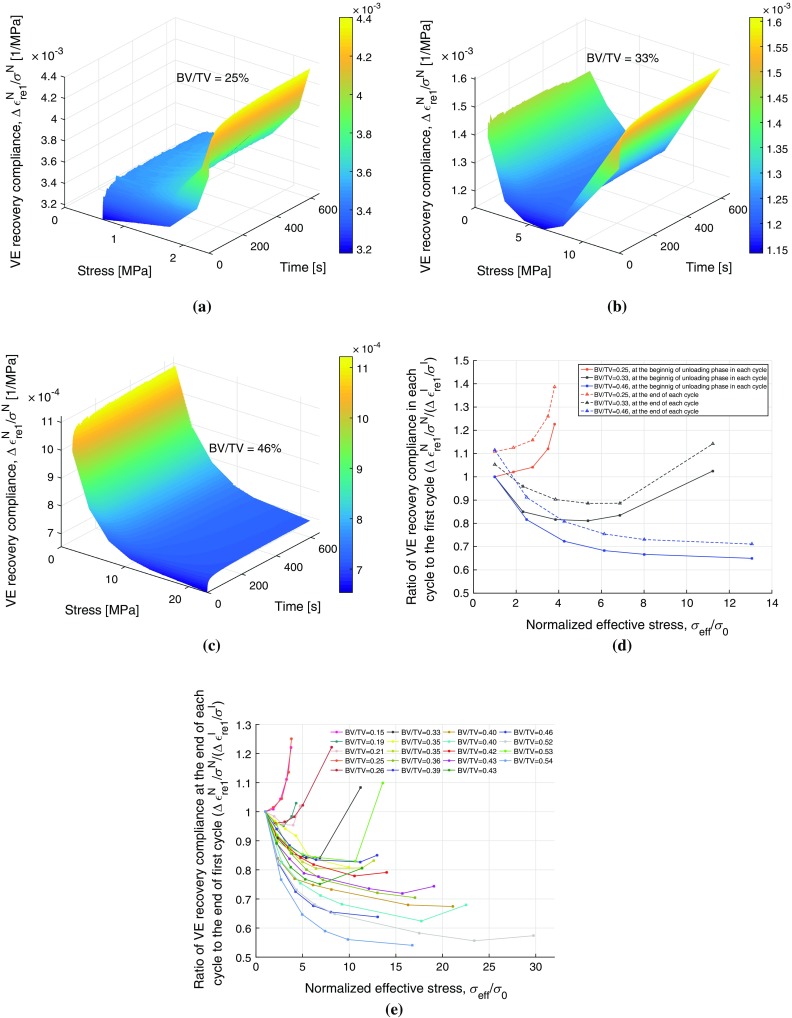
Experimental viscoelastic recovery compliance with the time and stress for samples: **a** S25 (BV/TV = 0.25), **b** S33 (BV/TV = 0.33), and **c** S46 (BV/TV = 0.46); **d** the ratio between the viscoelastic recovery compliance and the respective instantaneous compliance for each of the three samples plotted plotted against normalized effective stress, and **e** the ratio of viscoelastic recovery compliance at the end of each cycle to the respective value at the end of first cycle plotted against normalized effective stress for all 19 samples. Purely recoverable response was obtained from ${\Delta }\epsilon _\mathrm{re1}^N$ in each loading cycle

### Viscoelastic recovery compliance

The viscoelastic recovery compliance was evaluated in all cycles using ${\Delta }\epsilon _\mathrm{re1}^N/\sigma ^N$ (note that the numerator does not include irrecoverable strains) for all samples. Typical variation of compliance with time as well as with varying applied stress is shown in Fig. 4a–d for samples S25, S33 and S46. The units for compliance are 1/MPa. In the first loading cycle, for the three typical samples, the viscoelastic recovery compliance increased by 11 % (from $3.17\times 10^{-3}$ to $3.51\times 10^{-3}$), 6 % (from $1.40\times 10^{-3}$ to $1.48\times 10^{-3}$) and 12 % (from $1.00\times 10^{-3}$ to $1.12\times 10^{-3}$) at 600 s (end of unloading phase) for samples S25, S33 and S46, respectively (Fig. 4). Compliance was found to increase with time in all loading cycles as expected in viscoelastic material. However, the compliance for trabecular bone also found to vary with stress indicating a nonlinear viscoelastic response. For sample S25, the compliance increased from $3.51\times 10^{-3}$ at the end of cycle I to $4.40\times 10^{-3}$ at the end of cycle V. For high-density sample S46, the compliance decreased from $1.12\times 10^{-3}$ at the end of cycle I to $0.71\times 10^{-3}$ at the end of cycle VI. But in the sample S33, the compliance was found to first decrease from $1.48\times 10^{-3}$ at the end of cycle I to $1.25\times 10^{-3}$ at the end of loading cycle IV and then increase to $1.70\times 10^{-3}$ at the end of cycle VII. This stress-dependent compliance behaviour is shown in Fig. 4d for the three samples. Figure 4e shows that compliance increases with stress for low BV/TV samples, decreases with stress for high BV/TV samples, and first decreases with stress and then increases with stress for mid-BV/TV samples.

### Nonlinear viscoelastic parameters

**Fig. 5:**
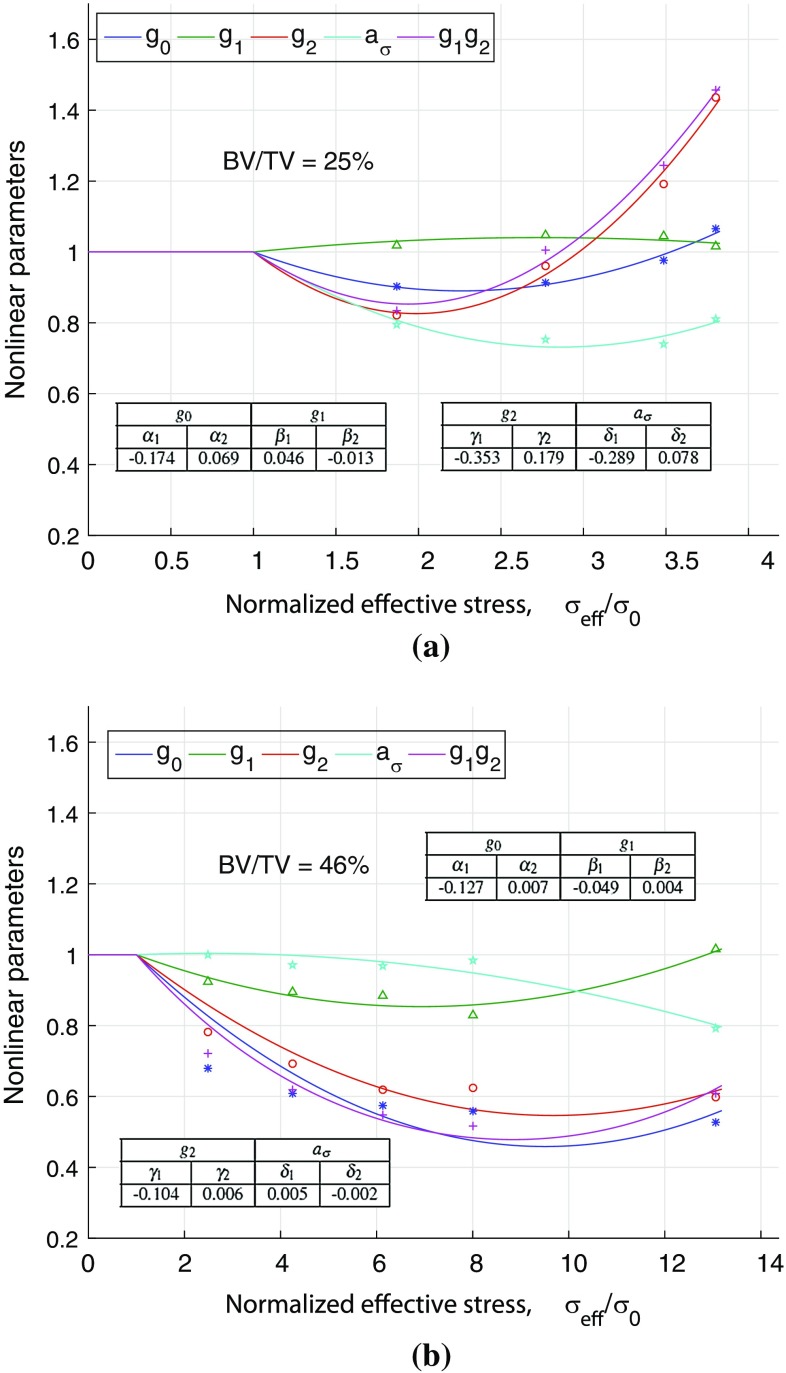
Nonlinear viscoelastic parameters, $g_0$, $g_1$, $g_2$ and $a_\sigma$, expressed as second-order polynomial functions of effective stress (Eqs. 19–22), are plotted against normalized effective stress for two samples with **a** BV/TV = 0.25 and **b** BV/TV = 0.46

The stress-dependent nonlinear viscoelastic parameters, $g_0$, $g_1$, $g_2$ and $a_{\sigma }$, were evaluated for all 19 samples. Fig. 5a, b shows the variation in these parameters for samples S25 and S46, respectively. The procedure assumes linear viscoelasticity in the first cycle (initial apparent strain of 0.2 %). Numerical values of stress-dependent nonlinear viscoelastic parameters along with other evaluated values are presented in Table 1 for all 19 samples. The results show that for sample S25, the values of $g_0$, $g_2$ and $a_{\sigma }$ first decrease and then increase with the stress level, whereas the value of $g_1$ first increases slightly and then decreases slightly with the stress level (Fig. 5a). The product of $g_1g_2$ which affects the transient response was also found to first decrease and then increase. These observations led us to the choice of a second-order polynomial function to represent the nonlinear VE parameters as functions of effective stress. These second-order functions produced coefficients of determination of $r^2$ = 0.97, 0.72, 0.98 and 0.69 for parameters $g_0$, $g_1$, $g_2$ and $a_{\sigma }$, respectively, as shown in Fig. 5a.

For sample S46, Fig. 5b, the parameters $g_0$, $g_1$ and $g_2$ were found to decrease and then increase with the stress level, and $a_{\sigma }$ was almost constant ($\approx$1) and then decreased in the last stress cycle. The second-order polynomial functions of effective stress produced $r^2$ values of 0.83, 0.90, 0.92 and 0.93 for $g_0$, $g_1$, $g_2$ and $a_{\sigma }$ , respectively, for sample S46. The increase in the values of $g_0$, $g_1$, $g_2$ or the product of $g_1g_2$ essentially means that the trabecular bone material experiences viscoelastic softening (reduction of stiffness) and decrease of these parameters imply that the material experiences stiffening.

**Fig. 6:**
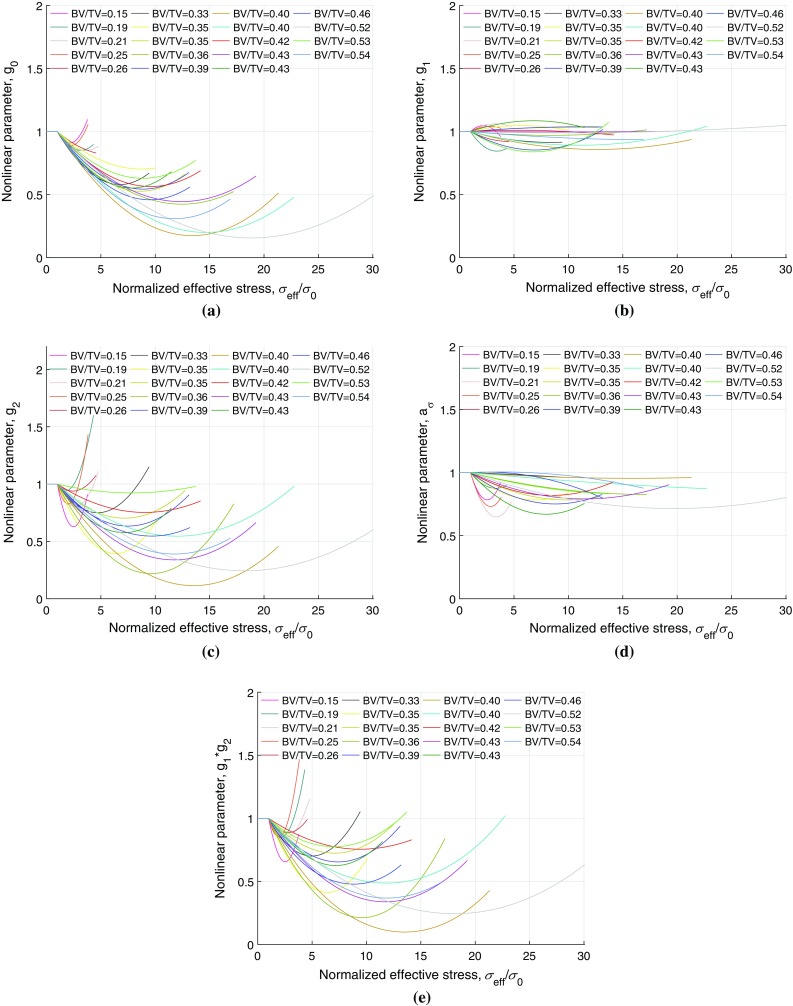
Nonlinear VE parameters, expressed as second-order polynomial functions of effective stress, for all 19 samples are plotted against normalized stress, **a** parameter $g_0$, **b** parameter $g_1$, **c** parameter $g_2$, **d** parameter $a_{\sigma }$, and **e** product of the parameters $g_1$ and $g_2$

Figure. 6a–d shows the variation of nonlinear VE parameters, $g_0$, $g_1$, $g_2$ and $a_\sigma$, respectively, which were expressed as polynomial functions of effective stress, for all samples. It can be seen that the variation described for two typical samples is largely followed by all.

### Irrecoverable strains

**Fig. 7:**
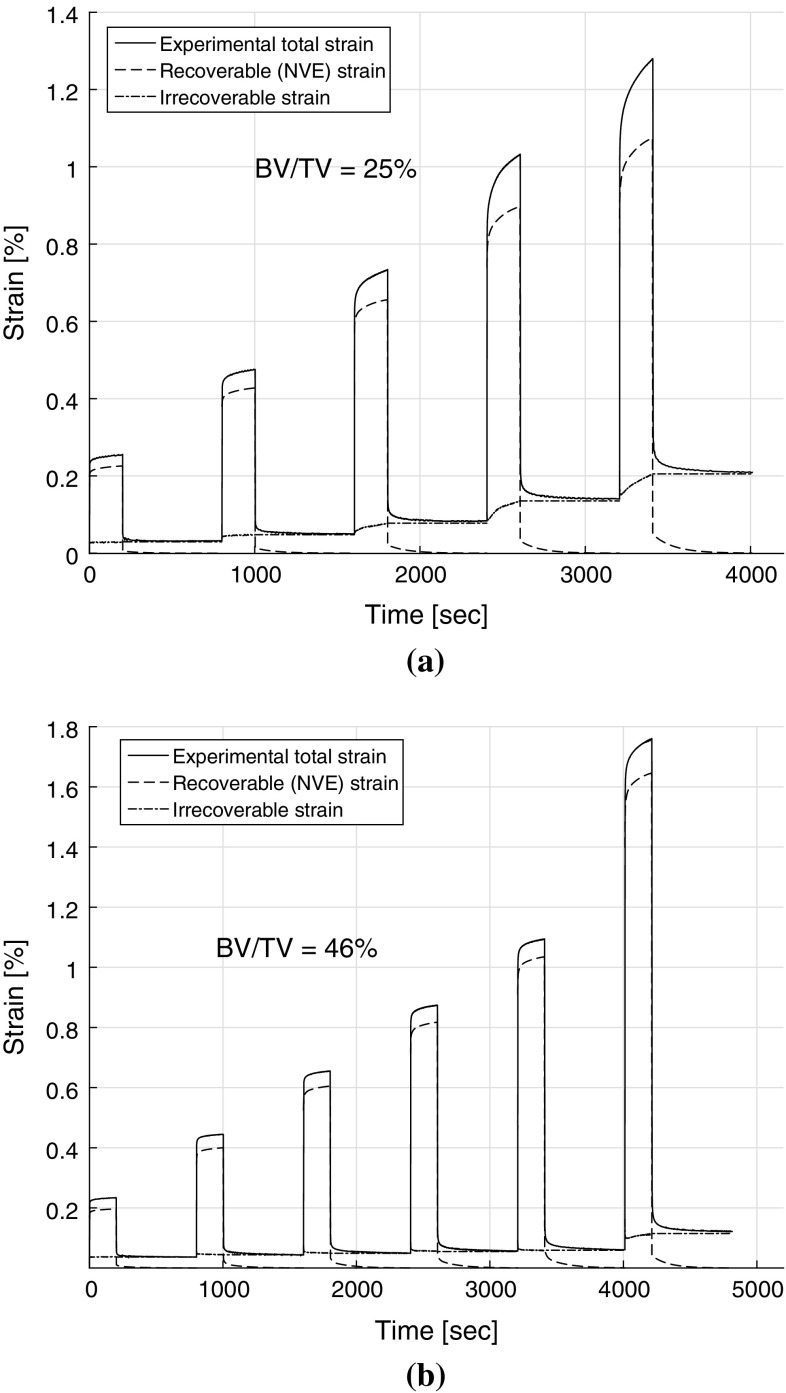
Pure viscoelastic and the irrecoverable strain responses are plotted along with the total creep strain response for two typical samples S25 and S46, **a** BV/TV = 0.25 and **b** BV/TV = 0.46, respectively

**Fig. 8:**
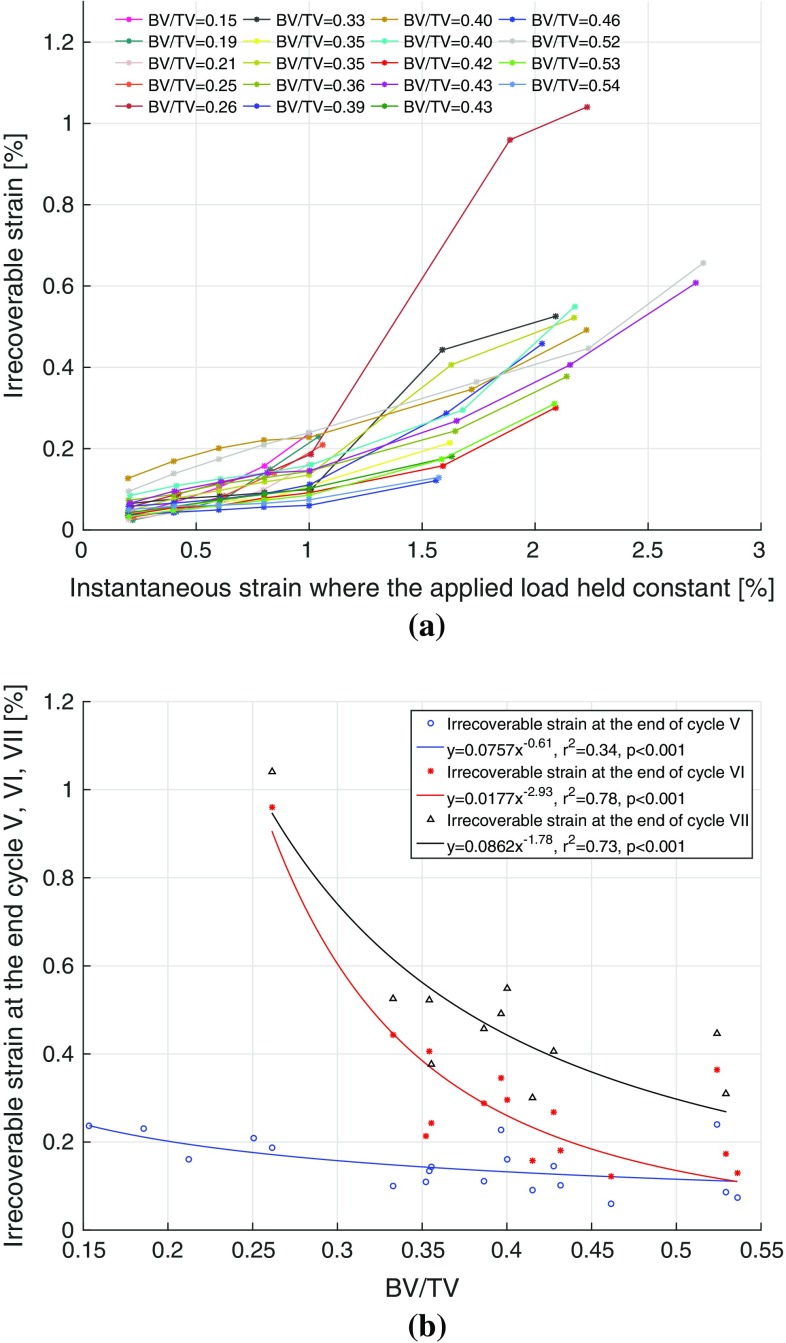
**a** Irrecoverable strains at the end of each loading cycle in each sample with the applied static strain (where plateau force was held constant during creep–recovery test), **b** irrecoverable strains in cycles V, VI and VII corresponding to static strains of 1.0, 1.5 and 2.0 % are plotted against BV/TV of all samples

The irrecoverable strain along with nonlinear viscoelastic (recoverable) strain response for samples S25 and S46 is shown in Fig. 7a, b. The figures also show the measured experimental strain response which comprises of the recoverable and irrecoverable strain components (Eq. 1). The viscoelastic strain was found to recover fully (below $7\,\upmu \epsilon$) in under 10 min during the recovery phase of each loading cycle. Irrecoverable strains exist even at the end of the first loading cycle (stress level corresponding to strain of 0.2 %) and were found to increase with stress. For sample S25, the irrecoverable strain increased to 0.20 % by the end of cycle V from 0.03 % in cycle I, Fig. 7a, whereas for sample S46, it increased to 0.12 % by the end of loading cycle VI from 0.03 % in cycle I, Fig. 7b. The irrecoverable strains in each loading cycle for all 19 samples are shown in Fig. 8a.

There were no significant correlations found between the irrecoverable strains and BV/TV in the loading cycles I–IV. However, a weak but significant power law correlation ($y=0.0757x^{-0.61}$, $r^2=0.34$, $p<0.001$) in the cycle V with BV/TV was found. At loading cycles at higher stress, strong and significant power law relationships $y=0.0177x^{-2.93}$ ($r^2=0.78$, $p<0.001$) and $y=0.0862x^{-1.78}$ ($r^2=0.73$, $p<0.001$) were found between the irrecoverable strains and BV/TV in the cycles VI and VII, respectively.

## Discussion

This study developed a novel methodology to evaluate time-dependent properties of trabecular bone. Our creep–recovery experiments at multiple stress levels demonstrate that the response of trabecular bone to mechanical forces is time-dependent and the strain always comprises of recoverable and irrecoverable components even at low stress levels. Our results show that the viscoelastic behaviour of trabecular bone varies nonlinearly with the applied stress.

Stress dependence of creep response has been previously examined in studies on polymers and concretes (Lai and Bakker 1995; Huang et al. 2011). In these studies, the creep–recovery tests were performed independently and involved long relaxation periods between stress cycles. We performed creep and recovery tests at varying load levels continuously without resting the sample in between the tests. We chose this protocol, as it was not apparent how long different trabecular bone samples would take to fully recover from any loading cycle. The adopted methodology required the residual strains from the previous cycle to be taken into account when evaluating the response of the following loading cycle.

The identification of viscoelastic parameters constitutes a two-step process. In the first step, the Prony coefficients associated with linear viscoelastic response are determined for the loading cycle at the lowest stress level, and in the second step, the linear viscoelastic response with additional appropriate constitutive parameters is manipulated to match-up with the experimental response at multiple stress levels using nonlinear least squares minimization technique; thereby, the corresponding constitutive parameters are evaluated at multiple load levels. A major strength of our methodology is that it permits separation of the recoverable response from the total strain response through the use of creep and recovery parts of the curves in each loading cycle. Thus, it is possible to assess accurately the viscoelastic response of trabecular bone. Linear viscoelastic properties were characterized by the Prony series based on the generalized three-term Kelvin model at the lowest stress cycle (corresponding to 0.2 % of applied static strain), assuming bone behaves linearly at this small strain. The nonlinear viscoelastic parameters were successfully fitted to polynomial functions which represent the parameters as continuous functions of stress levels. Previous studies have also reported that the time-dependent behaviour of the trabecular bone is nonlinear (Deligianni et al. 1994; Bowman et al. 1994; Yamamoto et al. 2006; Quaglini et al. 2009).

**Table 1:** Nonlinear VE parameters along with linear Prony coefficients and irrecoverable strains at multiple stress levels for all 19 samples

BV/TV	Linear Prony coefficients at $\sigma ^\mathrm{I}$	Cycle no.	$\epsilon _\mathrm{static} (\%)$	$\sigma ^{N}$ (MPa)	Nonlinear VE parameters				$\epsilon _\mathrm{irrec}$ (%)
					$g_{0}$	$g_{1}$	$g_{2}$	$a_{\sigma }$	
0.15	$$\left[ \begin{array}{c} D_{0} \\ D_{1} \\ D_{2} \\ D_{3} \\ \lambda _{1} \\ \lambda _{2} \\ \lambda _{3} \end{array}\right] =\left[ \begin{array}{c} 6.40\times 10^{-3}\\ 5.48\times 10^{-4} \\ 3.24\times 10^{-4} \\ 2.97\times 10^{-4} \\ 8.64\times 10^{-3} \\ 8.64\times 10^{-1} \\ 9.31\times 10^{-2} \end{array}\right] $$	I	0.20	0.36	1.00	1.00	1.00	1.00	0.041
		II	0.40	0.66	0.91	1.06	0.59	0.78	0.067
		III	0.60	0.94	0.94	1.03	0.67	0.82	0.104
		IV	0.80	1.17	0.99	1.01	0.82	0.85	0.158
		V	1.00	1.35	1.10	0.96	0.84	0.91	0.237
0.19	$$\left[ \begin{array}{c} D_{0}\\ D_{1} \\ D_{2} \\ D_{3} \\ \lambda _{1} \\ \lambda _{2} \\ \lambda _{3} \end{array}\right] =\left[ \begin{array}{c} 3.44\times 10^{-3}\\ 1.85\times 10^{-4} \\ 1.25\times 10^{-4} \\ 2.47\times 10^{-4} \\ 6.51\times 10^{-1} \\ 4.12\times 10^{-2} \\ 3.57\times 10^{-3} \end{array}\right] $$	I	0.20	0.64	1.00	1.00	1.00	1.00	0.024
		II	0.40	1.24	0.89	0.85	0.94	0.88	0.045
		III	0.60	1.89	0.87	0.89	1.02	0.92	0.076
		IV	0.80	2.44	0.85	0.86	1.50	0.86	0.150
		V	1.00	2.74	0.90	0.85	1.51	0.90	0.230
0.21	$$\left[ \begin{array}{c} D_{0} \\ D_{1} \\ D_{2} \\ D_{3} \\ \lambda _{1} \\ \lambda _{2} \\ \lambda _{3} \end{array}\right] =\left[ \begin{array}{c} 3.42\times 10^{-3}\\ 3.39\times 10^{-4}\\ 3.29\times 10^{-4} \\ 1.64\times 10^{-4} \\ 6.20\times 10^{-3} \\ 2.42\times 10^{+0} \\ 1.12\times 10^{-1} \end{array}\right] $$	I	0.20	0.60	1.00	1.00	1.00	1.00	0.026
		II	0.40	1.16	0.90	1.05	0.84	0.69	0.041
		III	0.60	1.73	0.87	1.06	0.82	0.69	0.062
		IV	0.80	2.38	0.85	1.05	0.91	0.73	0.099
		V	1.00	2.82	0.88	1.04	1.11	0.73	0.161
0.25	$$\left[ \begin{array}{c} D_{0}\\ D_{1} \\ D_{2} \\ D_{3} \\ \lambda _{1} \\ \lambda _{2} \\ \lambda _{3} \end{array}\right] =\left[ \begin{array}{c} 3.52\times 10^{-3} \\ 1.31\times 10^{-4} \\ 2.63\times 10^{-4} \\ 1.30\times 10^{-4} \\ 7.57\times 10^{-2} \\ 6.44\times 10^{-3} \\ 5.68\times 10^{-1} \end{array}\right] $$	I	0.20	0.64	1.00	1.00	1.00	1.00	0.032
		II	0.40	1.20	0.90	1.02	0.82	0.79	0.049
		III	0.60	1.77	0.91	1.05	0.96	0.75	0.084
		IV	0.80	2.23	0.98	1.04	1.19	0.74	0.140
		V	1.00	2.43	1.06	1.01	1.44	0.81	0.209
0.26	$$\left[ \begin{array}{c} D_{0} \\ D_{1} \\ D_{2} \\ D_{3} \\ \lambda _{1} \\ \lambda _{2} \\ \lambda _{3} \end{array}\right] =\left[ \begin{array}{c} 2.68\times 10^{-3} \\ 1.75\times 10^{-4} \\ 1.33\times 10^{-4} \\ 1.66\times 10^{-4} \\ 7.77\times 10^{-3} \\ 1.15\times 10^{-1} \\ 1.06\times 10^{+0} \end{array}\right] $$	I	0.20	0.80	1.00	1.00	1.00	1.00	0.057
		II	0.40	1.65	0.78	0.94	0.64	0.91	0.089
		III	0.60	2.48	0.77	0.99	0.71	0.88	0.116
		IV	0.80	3.28	0.81	0.90	0.65	0.96	0.142
		V	1.00	4.01	0.83	0.89	0.79	0.97	0.186
		VI	1.50	6.50	0.82	1.01	1.86	0.86	0.960
		VII	2.00	3.62	1.02	0.94	2.14	0.96	1.041
0.33	$$\left[ \begin{array}{c} D_{0} \\ D_{1} \\ D_{2} \\ D_{3} \\ \lambda _{1} \\ \lambda _{2} \\ \lambda _{3} \end{array}\right] =\left[ \begin{array}{c} 1.75\times 10^{-3} \\ 7.46\times 10^{-5} \\ 1.11\times 10^{-4} \\ 6.68\times 10^{-5} \\ 9.87\times 10^{-3} \\ 1.02\times 10^{+0} \\ 1.21\times 10^{-1} \end{array}\right] $$	I	0.20	1.19	1.00	1.00	1.00	1.00	0.065
		II	0.40	2.76	0.66	0.93	0.84	0.98	0.076
		III	0.60	4.58	0.63	0.94	0.74	0.99	0.083
		IV	0.80	6.40	0.62	0.92	0.71	0.98	0.091
		V	1.00	8.18	0.62	0.95	0.67	0.99	0.100
		VI	1.50	13.37	0.75	0.92	1.32	0.95	0.442
		VII	2.00	11.13	0.78	0.92	1.53	0.96	0.526
0.35	$$\left[ \begin{array}{c} D_{0} \\ D_{1} \\ D_{2} \\ D_{3} \\ \lambda _{1} \\ \lambda _{2} \\ \lambda _{3} \end{array}\right] =\left[ \begin{array}{c} 1.60\times 10^{-3} \\ 1.14\times 10^{-4} \\ 6.45\times 10^{-5} \\ 8.35\times 10^{-5} \\ 7.64\times 10^{-3} \\ 9.41\times 10^{-2} \\ 7.05\times 10^{-1} \end{array}\right] $$	I	0.20	1.31	1.00	1.00	1.00	1.00	0.039
		II	0.40	2.69	0.84	1.14	0.71	0.67	0.057
		III	0.60	4.09	0.84	1.08	0.60	0.78	0.072
		IV	0.80	5.59	0.82	1.00	0.57	0.87	0.075
		V	1.00	7.50	0.78	1.00	0.37	0.93	0.109
		VI	1.50	13.01	0.70	1.02	0.66	0.80	0.214
0.35	$$\left[ \begin{array}{c} D_{0} \\ D_{1} \\ D_{2} \\ D_{3} \\ \lambda _{1} \\ \lambda _{2} \\ \lambda _{3} \end{array}\right] =\left[ \begin{array}{c} 2.16\times 10^{-3} \\ 1.41\times 10^{-4} \\ 1.43\times 10^{-4} \\ 1.11\times 10^{-4} \\ 6.41\times 10^{-3} \\ 1.41\times 10^{+0} \\ 1.22\times 10^{-1} \end{array}\right] $$	I	0.20	0.94	1.00	1.00	1.00	1.00	0.047
		II	0.40	2.16	0.70	1.02	0.84	0.84	0.077
		III	0.60	3.46	0.67	1.03	0.80	0.85	0.097
		IV	0.80	4.67	0.65	1.02	0.75	0.86	0.118
		V	1.00	6.04	0.63	1.02	0.72	0.87	0.135
		VI	1.50	10.67	0.62	1.04	0.82	0.80	0.406
		VII	2.00	11.83	0.62	1.03	0.94	0.79	0.522
0.36	$$\left[ \begin{array}{c} D_{0} \\ D_{1} \\ D_{2} \\ D_{3} \\ \lambda _{1} \\ \lambda _{2} \\ \lambda _{3} \end{array}\right] =\left[ \begin{array}{c} 2.07\times 10^{-3} \\ 1.48\times 10^{-4} \\ 1.52\times 10^{-4} \\ 1.55\times 10^{-4} \\ 1.63\times 10^{-1} \\ 1.07\times 10^{-2} \\ 1.75\times 10^{+0} \end{array}\right] $$	I	0.20	0.98	1.00	1.00	1.00	1.00	0.073
		II	0.40	2.12	0.71	1.20	0.45	0.70	0.087
		III	0.60	3.67	0.65	1.02	0.43	0.87	0.112
		IV	0.80	5.28	0.62	0.95	0.41	0.93	0.128
		V	1.00	7.02	0.59	0.89	0.40	0.97	0.144
		VI	1.50	12.73	0.54	1.00	0.42	0.89	0.244
		VII	2.00	16.68	0.45	1.01	0.75	0.79	0.377
0.39	$$\left[ \begin{array}{c} D_{0} \\ D_{1} \\ D_{2} \\ D_{3} \\ \lambda _{1} \\ \lambda _{2} \\ \lambda _{3} \end{array}\right] =\left[ \begin{array}{c} 1.53\times 10^{-3} \\ 1.07\times 10^{-4} \\ 1.07\times 10^{-4} \\ 8.45\times 10^{-5} \\ 6.37\times 10^{-3} \\ 1.27\times 10^{+0} \\ 1.23\times 10^{-1} \end{array}\right] $$	I	0.20	1.33	1.00	1.00	1.00	1.00	0.058
		II	0.40	2.92	0.76	0.83	0.76	0.97	0.066
		III	0.60	4.79	0.67	1.02	0.78	0.83	0.076
		IV	0.80	6.69	0.63	1.05	0.83	0.75	0.089
		V	1.00	8.53	0.65	1.07	0.64	0.79	0.111
		VI	1.50	14.81	0.66	1.02	0.56	0.86	0.288
		VII	2.00	17.19	0.60	1.04	1.01	0.77	0.458
0.40	$$\left[ \begin{array}{c} D_{0} \\ D_{1} \\ D_{2} \\ D_{3} \\ \lambda _{1} \\ \lambda _{2} \\ \lambda _{3} \end{array}\right] =\left[ \begin{array}{c} 2.88\times 10^{-3} \\ 2.36\times 10^{-4} \\ 5.01\times 10^{-4} \\ 2.56\times 10^{-4} \\ 1.12\times 10^{-2} \\ 2.57\times 10^{+0} \\ 1.54\times 10^{-1} \end{array}\right] $$	I	0.20	0.71	1.00	1.00	1.00	1.00	0.127
		II	0.40	1.65	0.46	0.89	0.51	0.97	0.170
		III	0.60	2.95	0.44	0.87	0.41	0.96	0.201
		IV	0.80	4.32	0.43	0.90	0.40	0.98	0.220
		V	1.00	5.74	0.43	0.91	0.34	0.99	0.227
		VI	1.50	11.56	0.39	0.92	0.36	0.94	0.346
		VII	2.00	14.98	0.39	0.90	0.33	0.97	0.491
0.40	$$\left[ \begin{array}{c} D_{0} \\ D_{1} \\ D_{2} \\ D_{3} \\ \lambda _{1} \\ \lambda _{2} \\ \lambda _{3} \end{array}\right] =\left[ \begin{array}{c} 2.69\times 10^{-3} \\ 9.10\times 10^{-5} \\ 1.02\times 10^{-4} \\ 1.26\times 10^{-4} \\ 1.55\times 10^{-1} \\ 9.68\times 10^{-3} \\ 1.13\times 10^{+0} \end{array}\right] $$	I	0.20	0.77	1.00	1.00	1.00	1.00	0.085
		II	0.40	2.13	0.52	0.85	0.73	0.96	0.109
		III	0.60	3.69	0.47	0.88	0.67	0.98	0.126
		IV	0.80	5.35	0.43	0.96	0.70	0.91	0.141
		V	1.00	7.11	0.43	0.88	0.60	0.98	0.160
		VI	1.50	13.69	0.37	0.99	0.69	0.89	0.295
		VII	2.00	17.41	0.38	1.01	0.95	0.87	0.550
0.42	$$\left[ \begin{array}{c} D_{0} \\ D_{1} \\ D_{2} \\ D_{3} \\ \lambda _{1} \\ \lambda _{2} \\ \lambda _{3} \end{array}\right] =\left[ \begin{array}{c} 1.47\times 10^{-3} \\ 1.09\times 10^{-4} \\ 8.72\times 10^{-5} \\ 7.91\times 10^{-5} \\ 2.81\times 10^{+0}\\ 8.63\times 10^{-3} \\ 1.76\times 10^{-1} \end{array}\right] $$	I	0.20	1.37	1.00	1.00	1.00	1.00	0.037
		II	0.40	2.97	0.73	1.03	0.98	0.83	0.054
		III	0.60	4.74	0.71	1.04	0.86	0.82	0.059
		IV	0.80	6.57	0.69	1.04	0.82	0.84	0.079
		V	1.00	8.44	0.66	1.03	0.85	0.85	0.091
		VI	1.50	14.45	0.67	0.91	0.68	0.96	0.158
		VII	2.00	19.20	0.63	1.01	0.88	0.86	0.301
0.43	$$\left[ \begin{array}{c} D_{0} \\ D_{1} \\ D_{2} \\ D_{3} \\ \lambda _{1} \\ \lambda _{2} \\ \lambda _{3} \end{array}\right] =\left[ \begin{array}{c} 1.94\times 10^{-3} \\ 1.19\times 10^{-4} \\ 1.75\times 10^{-4} \\ 9.27\times 10^{-5} \\ 7.85\times 10^{-1} \\ 7.38\times 10^{-3} \\ 9.59\times 10^{-2} \end{array}\right] $$	I	0.20	1.08	1.00	1.00	1.00	1.00	0.066
		II	0.40	2.39	0.67	1.09	0.60	0.74	0.096
		III	0.60	3.88	0.63	1.03	0.59	0.80	0.118
		IV	0.80	5.54	0.60	1.05	0.55	0.77	0.141
		V	1.00	7.22	0.61	0.89	0.52	0.96	0.146
		VI	1.50	13.04	0.57	1.01	0.42	0.84	0.268
		VII	2.00	16.91	0.55	1.00	0.51	0.85	0.406
		VIII	2.50	20.56	0.56	1.00	0.57	0.86	0.608
0.43	$$\left[ \begin{array}{c} D_{0} \\ D_{1} \\ D_{2} \\ D_{3} \\ \lambda _{1} \\ \lambda _{2} \\ \lambda _{3} \end{array}\right] =\left[ \begin{array}{c} 9.40\times 10^{-4} \\ 3.67\times 10^{-5} \\ 6.46\times 10^{-5} \\ 6.43\times 10^{-5} \\ 1.06\times 10^{-1} \\ 6.74\times 10^{-3} \\ 9.59\times 10^{-1} \end{array}\right] $$	I	0.20	2.13	1.00	1.00	1.00	1.00	0.042
		II	0.40	4.75	0.74	1.09	0.70	0.73	0.057
		III	0.60	7.96	0.67	1.08	0.64	0.70	0.074
		IV	0.80	11.29	0.64	1.07	0.62	0.75	0.088
		V	1.00	14.65	0.61	1.06	0.68	0.78	0.102
		VI	1.50	24.26	0.66	1.04	0.75	0.72	0.180
0.46	$$\left[ \begin{array}{c} D_{0} \\ D_{1} \\ D_{2} \\ D_{3} \\ \lambda _{1} \\ \lambda _{2} \\ \lambda _{3} \end{array}\right] =\left[ \begin{array}{c} 1.16\times 10^{-3} \\ 4.19\times 10^{-5} \\ 5.82\times 10^{-5} \\ 8.91\times 10^{-5} \\ 6.99\times 10^{-2} \\ 6.48\times 10^{-3} \\ 6.75\times 10^{-1} \end{array}\right] $$	I	0.20	1.75	1.00	1.00	1.00	1.00	0.037
		II	0.40	4.38	0.68	0.92	0.78	1.00	0.043
		III	0.60	7.45	0.61	0.89	0.69	0.97	0.049
		IV	0.80	10.77	0.57	0.88	0.62	0.97	0.056
		V	1.00	14.06	0.56	0.83	0.62	0.98	0.060
		VI	1.50	22.92	0.53	1.01	0.60	0.79	0.121
0.52	$$\left[ \begin{array}{c} D_{0} \\ D_{1} \\ D_{2} \\ D_{3} \\ \lambda _{1} \\ \lambda _{2} \\ \lambda _{3} \end{array}\right] =\left[ \begin{array}{c} 2.29\times 10^{-3} \\ 1.74\times 10^{-4} \\ 2.03\times 10^{-4} \\ 1.60\times 10^{-4} \\ 1.50\times 10^{+0} \\ 6.85\times 10^{-3} \\ 1.29\times 10^{-1} \end{array}\right] $$	I	0.20	0.89	1.00	1.00	1.00	1.00	0.095
		II	0.40	2.25	0.48	1.13	0.63	0.66	0.138
		III	0.60	3.87	0.43	1.09	0.60	0.69	0.175
		IV	0.80	5.62	0.42	1.08	0.49	0.74	0.210
		V	1.00	7.54	0.43	0.76	0.50	0.97	0.239
		VI	1.50	15.62	0.36	1.05	0.41	0.76	0.364
		VII	2.00	20.88	0.36	1.03	0.32	0.82	0.447
		VIII	2.50	26.56	0.33	1.03	0.53	0.73	0.656
0.53	$$\left[ \begin{array}{c} D_{0} \\ D_{1} \\ D_{2} \\ D_{3} \\ \lambda _{1} \\ \lambda _{2} \\ \lambda _{3} \end{array}\right] =\left[ \begin{array}{c} 9.05\times 10^{-4} \\ 4.26\times 10^{-5} \\ 3.35\times 10^{-5} \\ 4.21\times 10^{-5} \\ 6.32\times 10^{-1} \\ 6.40\times 10^{-2} \\ 5.54\times 10^{-3} \end{array}\right] $$	I	0.20	2.22	1.00	1.00	1.00	1.00	0.033
		II	0.40	5.03	0.79	0.81	0.95	0.92	0.048
		III	0.60	8.02	0.75	0.84	0.88	0.92	0.059
		IV	0.80	11.05	0.73	0.83	0.90	0.94	0.073
		V	1.00	14.10	0.71	0.87	0.91	0.96	0.085
		VI	1.50	23.66	0.67	1.00	1.07	0.78	0.174
		VII	2.00	30.13	0.75	1.01	0.90	0.86	0.310
0.54	$$\left[ \begin{array}{c} D_{0} \\ D_{1} \\ D_{2} \\ D_{3} \\ \lambda _{1} \\ \lambda _{2} \\ \lambda _{3} \end{array}\right] =\left[ \begin{array}{c} 1.36\times 10^{-3} \\ 8.02\times 10^{-5} \\ 6.44\times 10^{-5} \\ 6.17\times 10^{-5} \\ 8.56\times 10^{-1} \\ 8.64\times 10^{-3} \\ 9.62\times 10^{-2} \end{array}\right] $$	I	0.20	1.49	1.00	1.00	1.00	1.00	0.050
		II	0.40	4.00	0.58	1.06	0.71	1.00	0.058
		III	0.60	7.38	0.50	1.11	0.48	1.00	0.061
		IV	0.80	11.01	0.45	0.90	0.60	0.98	0.065
		V	1.00	14.66	0.45	0.87	0.47	1.00	0.074
		VI	1.50	24.90	0.42	0.96	0.49	0.88	0.129

BV/TV is the bone volume fraction, $D_0$ is the instantaneous compliance in 1/MPa, $D_n$ ($n = 1, 2, 3$) are transient compliance coefficients in 1/MPa, and $\lambda _n$ ($n = 1, 2, 3$) are reciprocal of *n*th retardation time in Prony series in $s^{-1}$, $\epsilon _\mathrm{static}$ is the applied static strain in each loading cycle, and $\sigma ^N$ is the stress corresponding to plateau stress in the *N*th loading cycle in MPa. Parameters $g_{0}$, $g_{1}$, $g_{2}$, $a_{\sigma }$ are stress-dependent nonlinear VE parameters and $\epsilon _\mathrm{irrec}$ is the irrecoverable strain exist at the end of each loading cycle

The viscoelastic recovery compliance was found to vary with time as well as with the applied stress demonstrating the nonlinear stress-dependent viscoelastic response of trabecular bone (Fig. 4). The samples with medium BV/TV (e.g. S33, Fig. 4b) show an initially decreasing and then increasing viscoelastic recovery compliance with increasing stress. This indicates that the sample first becomes stiffer and then experiences softening (stiffness degradation). This could be due to the reorganization of the micro- or ultrastructural components in the bone matrix to make it stiffer initially followed by localized buckling and/or damage of trabeculae causing softening. Nair et al. (2014) conducted compressive tests on mineralized and non-mineralized collagen microfibrils at molecular level at different compressive stress levels and found that the elastic modulus of mineralized collagen fibril increases significantly (stiffening) as the applied compressive load increases, whereas the non-mineralized samples showed reduced elastic modulus (higher deformability) with increase in load. Our study demonstrates that this stiffening at ultrastructural level translates to macro-level stiffening behaviour. Similarly, excessive deformation at molecular level may break the bonds between organic and inorganic phases which can result in microdamage which manifests itself as softening at the apparent level. In general, for low BV/TV samples, softening initiates at low stress levels (e.g. S25, Fig. 4a), whereas the high BV/TV samples indicate stiffening with little or no degradation even at the higher stress levels at which they were tested (Fig. 4c). Thus, micro-/ultrastructural reorganization and localized buckling and/or damage may make a varying contribution (with BV/TV playing an important role) to the apparent stiffening–softening behaviour with increasing stress. At higher strain levels, the collective effect of buckling and damage in the individual trabeculae will become dominant resulting in failure or tertiary creep. Previous studies have reported that the presence of marrow may also result in hydraulic stiffening (Cowin 1999) at higher strain rates. However, the unconfined MLCR experiments in our study were conducted at relatively low strain rates ($0.01\,\text {s}^{-1}$), and it is unlikely that marrow would have played a role in the observed stiffening phenomena. Kim et al. (2012) reported that the post-creep unloading modulus is significantly higher than pre-creep loading modulus indicating that the stiffening of trabecular bone occurs under compressive creep, and authors attributed this behaviour to the possible reorganization of micro- or ultrastructural components in the bone. Our study also found similar stiffening at first and then softening under compressive creep.

All samples showed similar convex shape (Fig. 6a) for parameter $g_0$, which affects the instantaneous response, depending on their BV/TV with the coefficients of determination ($r^2$) of the polynomial functions were in the range of 0.18–0.99. The product of the parameters $g_1$ and $g_2$ which affects the transient response, Fig. 6e, produced the $r^2$ value in the range of 0.37–0.99. Some of the second-order polynomial functions of $g_0$ and $g_1g_2$ for some samples were weakly correlated; however, all of the correlations were positive and showed an initially decreasing and then increasing trend, which implies decreasing and increasing trend in the instantaneous and transient responses (recoverable compliance), respectively, with increasing stress. These functions of stress-dependent parameters explain the stiffening–softening behaviour of trabecular bone well under compressive creep loading. The change in parameter $a_\sigma$ shows the nonlinearity in the time-shift factor as a function of stress. The approximations using second-order polynomial functions of stress were considered appropriate as we had only data points corresponding to 5–8 stress levels.

The outstanding fact about these approximations is that all the functions revealed a stiffening–softening behaviour for all trabecular bone samples with varying degrees of success. With increasing stress the parameter $g_0$ and the product $g_1g_2$ reduce to less than 1 indicating stiffening (or reduced compliance) followed by an increase beyond 1 indicating softening (or increased compliance) with the further increase in stress . This can be clearly seen Fig. 6, and it can be observed that the viscoelastic response of samples with lower BV/TV was significantly different from samples with higher BV/TV. In general, for lower BV/TV samples, the parameters reach their minima and increase to greater than 1 rapidly, indicating quicker stiffening–softening behaviour with stress. For samples with higher BV/TV, the same behaviour was observed to vary more slowly with stress. From our results, it appears BV/TV is a good predictor of nonlinear stress-dependent viscoelastic response of the trabecular bone.

Irrecoverable strains (Fig. 8a) were found to exist even at smaller load levels. These strains existed consistently in all the samples and were of similar magnitudes in their first loading cycles. We believe these strains occur due to the material being loaded to strains beyond its yield point in some localized regions and entering the realm of irreversible deformation. Kim et al. (2012) reported that the residual strain, which they defined as strain that remain at the end of the unloading phase, of $1797 \pm 1391\,\upmu \epsilon$ remained after 2 h of strain recovery in the unloading phase when the plateau force corresponding to static strain of $2000\,\upmu \epsilon$ was applied in a creep test. Yamamoto et al. (2006) also reported residual strains and found that their magnitude was of a similar magnitude to the applied static strain ($515 \pm 255\,\upmu \epsilon$ and $1565 \pm 590\mu \epsilon$ for applied static strains of 750$\mu \epsilon$ and $1500\,\upmu \epsilon$, respectively) at the end 35 h of recovery period. From this, they estimated that these residual strains will fully recover in 26–63 days. Our study concludes that these residual strains are, in fact, irrecoverable (permanent) strains and never recover in vitro. We applied plateau load only for 200 s, the resulting irrecoverable strain magnitudes at the end of unloading phase (600 s of strain recovery) were of the order of 242–$1267\,\upmu \epsilon$ in the first loading cycle where applied plateau load corresponds to static strain of $2000\,\upmu \epsilon$, consistent with those observed in the previous studies (Yamamoto et al. 2006; Kim et al. 2012). However, in vivo, since bone is a living tissue, microdamage (which is the cause of these permanent strains) is likely to be repaired and replaced by a newer bone material via remodelling. In fact, microdamage in bone acts as a stimulus for directing biological activity (Burr et al. 1985; Lee et al. 2002). The microdamage initiates at scales below the macroscopic porosity of the bone and may be affected by intrinsic viscoelasticity of the tissue phase. The newly formed material due to bone remodelling may have less mineral which may increase compliance locally. The overall viscoelastic response at apparent level represents an average of old and new bone.

Kim et al. (2012) also reported from their experimental creep tests that the loading creep rate (during plateau load) is significantly higher than the unloading creep rate (during strain recovery in unloading phase) in trabecular bone. This possibly indicates that the creep response during plateau loading contains evolution of not only recoverable strain, but also some irreversible strain response. Our study validates this phenomenon and concludes that the creep response of the trabecular bone always contains both recoverable and irrecoverable responses even at smaller strains/stresses.

These irrecoverable strains at lower loading cycles (I–IV) were found to have no correlation with BV/TV. However, as the applied plateau loads increase in the higher loading cycles (V–VII), these strains strongly depend on BV/TV, Fig. 8b. Samples with lower BV/TV experienced higher irreversible strains with power law relationships, and irreversible strains decreased with the increasing BV/TV at the same applied strain level, Fig. 8b.

The mechanisms driving the viscoelastic behaviour in trabecular bone are not yet completely understood. It has been speculated that the individual constituents at different hierarchical levels in the trabecular bone and its microstructure contribute to the viscoelastic behaviour at the specimen level. Linde (1994) pointed out that the viscoelastic response of trabecular bone may depend on both the presence of marrow within the tissue and properties of the tissue itself, and Bowman et al. (1999) suggested that the collagen phase is responsible for the creep behaviour of the trabecular bone. Nair et al. (2014) suggested that extrafibrillar mineralization is mandatory along with intrafibrillar mineralization to provide the required bone mechanical properties. Further investigations are required to explicitly quantify the contributions of individual constituents to the apparent level viscoelastic behaviour of bone. However, from our results, it is evident that the BV/TV plays a major role in predicting the apparent level viscoelastic behaviour (Manda et al. 2016).

This work can be incorporated in finite element (FE) programs by coding a user defined material (UMAT) subroutine based on Schapery’s single integral model (Schapery 1969), which is not generally available in commercial FE packages. The linear Prony coefficients and the stress-dependent nonlinear VE parameters reported in Table 1 will act as input to the UMAT. The nonlinear VE parameters need to be supplied as smooth functions of stress (Eqs. 19–22).

Our study also has a few limitations. Firstly, it is not possible in practice to perform ideal creep–recovery experiments, and in our tests, the time intervals during the ramp loading and unloading are finite (1 s to reach 1.0 % strain with the strain rate of $0.01\,\mathrm{s}^{-1}$). Small viscoelastic deformations are likely to occur during the ramp loading phase; it may be possible to include these in a more elaborate model. In this study, finite ramp loading/unloading was treated as instantaneous in our material model; we believe this assumption has negligible effect on the evaluated material parameters. Our creep tests were performed with the plateau load holding time of 200 s which we believe is sufficiently long in comparison with the ramp loading/unloading time; it will have a negligible effect on the measured creep response. As in many previous studies, our experiments were performed at room temperature. It is possible that increase in temperature to $37\,^{\circ }\mathrm{C}$ may have a small effect on the creep behaviour; currently, the published data to confirm or invalidate this are limited .
